# Endogenous GLP-1 as a key self-defense molecule against lipotoxicity in pancreatic islets

**DOI:** 10.3892/ijmm.2015.2207

**Published:** 2015-05-12

**Authors:** CHENGHU HUANG, LI YUAN, SHUYI CAO

**Affiliations:** Department of Endocrinology, Union Hospital, Tongji Medical College, Huazhong University of Science and Technology, Wuhan, Hubei 430022, P.R. China

**Keywords:** glucagon-like peptide-1, prohormone convertase 1/3, lipotoxicity, oxidative stress, inflammation, pancreatic islet

## Abstract

The number of pro-α cells is known to increase in response to β cell injury and these cells then generate glucagon-like peptide-1 (GLP-1), thus attenuating the development of diabetes. The aim of the present study was to further examine the role and the mechanisms responsible for intra-islet GLP-1 production as a self-protective response against lipotoxicity. The levels of the key enzyme, prohormone convertase 1/3 (PC1/3), as well as the synthesis and release of GLP-1 in models of lipotoxicity were measured. Furthermore, islet viability, apoptosis, oxidative stress and inflammation, as well as islet structure were assessed after altering GLP-1 receptor signaling. Both prolonged exposure to palmitate and a high-fat diet facilitated PC1/3 expression, as well as the synthesis and release of GLP-1 induced by β cell injury and the generation of pro-α cells. Prolonged exposure to palmitate increased reactive oxygen species (ROS) production, and the antioxidant, N-acetylcysteine (NAC), partially prevented the detrimental effects induced by palmitate on β cells, resulting in decreased GLP-1 levels. Furthermore, the inhibition of GLP-1 receptor (GLP-1R) signaling by treatment with exendin-(9-39) further decreased cell viability, increased cell apoptosis and caused a stronger inhibition of the β cell-specific transcription factor, pancreatic duodenal homeobox 1 (PDX1). Moreover, treatment with the GLP-1R agonist, liraglutide, normalized islet structure and function, resulting in a decrease in cell death and in the amelioration of β cell marker expression. Importantly, liraglutide maintained the oxidative balance and decreased inflammatory factor and p65 expression. Overall, our data demonstrate that an increase in the number of pro-α cells and the activation of the intra-islet GLP-1 system comprise a self-defense mechanism for enhancing β cell survival to combat lipid overload, which is in part mediated by oxidative stress and inflammation.

## Introduction

Generally, the ectopic overaccumulation of lipids can trigger cellular dysregulation and functional tissue impairment, a process referred to as lipotoxicity (1). The investigation of the molecular mechanisms involved in lipotoxicity strongly indicates that excess lipids impair cell functions, leading to metabolically relevant cellular dysfunction, inflammation and oxidative stress (2,3). The prolonged exposure of β cells to increased levels of fatty acids, a major characteristic of lipotoxicity, elicits decreased insulin secretion, compromised insulin gene expression and β cell apoptosis (2). Glucagon-like peptide-1 (GLP-1) is a gastrointestinal hormone primarily secreted by L cells in the intestine in response to food intake (4). Intriguingly, GLP-1 exhibits beneficial pleiotropic effects on β cells by binding to a specific receptor (GLP-1R) and enhancing β cell proliferation and survival (5,6). GLP-1 has also been shown to protect pancreatic β cells against lipotoxicity-induced apoptosis and promotes insulin gene expression (7–10). Moreover, the GLP-1R agonist, liraglutide, has been shown to suppress oxidative stress in rats with streptozotocin-induced diabetes (11). Importantly, intra-islet GLP-1 functions as a paracrine signal for damaged β cells and is involved in protecting and regenerating them (12). In addition, the GLP-1R agonist, exendin-4, inhibits human islet inflammation (13).

Recently, interest in GLP-1 has intensified due to numerous important discoveries revealing that a functional GLP-1 system resides in α cells and is responsive to β cell stress and injury (14,15). Under normal conditions, pancreatic α cells secrete proglucagon, which is cleaved into glucagon by prohormone convertase 2 (PC2). However, under β cell stress conditions, α cell hyperplasia, which is a hallmark of β cell injury, develops (16). Notably, the dedifferentiation of hyperplastic α cells in adult islets seems to be preserved as an immature, pro-α phenotype attributable to the expression of prohormone convertase 1/3 (PC1/3) (15), which is the key enzyme in the processing of proglucagon into GLP-1 peptides in α cells (17–20). Thus, we hypothesized that the activation of the intra-islet GLP-1 system may be a protective measure for enhancing cellular survival.

We thus hypothesized that an intra-islet GLP-1 system may be the direct target of signals and self-preservation mechanisms that enhance β cell survival against lipotoxicity, in which oxidative stress plays a critical role. Our findings suggest that an elevated number of immature pro-α cells and the generation of GLP-1 are an advantage to β cells during conditions of high metabolic demand or stress, and that this ‘self-defense’ behavior facilitates β cell survival by reshaping the oxidative balance and inhibiting inflammation.

## Materials and methods

### Animals

Male wild-type C57BL/6J mice were used for all the islet experiments. The mice were maintained under standard light conditions (12/12-h light/dark cycle) and were allowed free access to food and water. The male C57BL/6 mice and their food were purchased from Beijing HFK Bio-Technology Co., Ltd. (Beijing, China). The care and experimental treatment of the animals were approved by the Animal Research Committee of Tongji Medical College, Huazhong University of Science and Technology, Wuhan, China. Six-week-old mice, weighing 21±2 g, were randomized into groups and fed either a high-fat diet (HFD; 20% protein, 20% carbohydrate and 60% fat), or a standard rodent chow diet [low-fat diet (LFD); 20% protein, 70% carbohydrate and 10% fat]. After 4 weeks on the HFD or LFD, the mice were injected with liraglutide (200 mg/kg body weight; Novo Nordisk, Princeton, NJ, USA) or a placebo [phosphate-buffered saline (PBS)] daily for 4 weeks. Body weight and food intake were measured weekly. To exclude the differences induced by food intake, all the mice fed the HFD were pair-fed. After the 8 weeks of feeding and drug administration, the mice were anesthetized with an intraperitoneal injection of pentobarbital sodium (0.6 mg/kg body weight).

### Metabolic measurements

For the measurement of fasting blood glucagon and insulin levels, the mice were fasted 6 h. The insulin and glucagon concentrations were determined using the the insulin enzyme-linked immunosorbent assay (ELISA) kit (Millipore, Boston, MA, USA). The plasma GLP-1 concentrations were measured using an ELISA kit (Linco Research, St. Charles, MO, USA).

### Islet isolation and cell culture

Non-diabetic mouse islets were isolated from the pancreata according to a previously described method (21). Specifically, the pancreas was perfused through the common bile duct with 1.5 mg/ml collagenase P (Roche Applied Science, Indianapolis, IN, USA), incubated at room temperature, and then further separated from the acinar tissue using a Histopaque 1077 gradient. The islets were isolated by hand and cultured for 24–72 h in RPMI-1640 medium in a 5% CO_2_ incubator. Subsequently, the islets were examined according to the steps outlined below for the functional analysis.

### Functional analysis

The non-diabetic islets were seeded into 24-well plates; 25 islets were added to each well. The palmitate solution was prepared as previously described (22). Following incubation in Krebs-Ringer Bicarbonate (KRB) buffer with 5.6 mmol/l glucose for 1 h, the islets were incubated under the following conditions: 0.5% BSA (as a control) or 0.5 mmol/l palmitate bound to 0.5% BSA for 24, 48 or 72 h. We examined the role of oxidative stress in the detrimental effects of palmitate by treating the islets with the antioxidant, N-acetylcysteine (NAC) (5 mmol/l; Sigma-Aldrich, St. Louis, MO, USA). Finally, to examine the bioactivity of islet-released GLP-1, experiments were performed in the absence or presence of the GLP-1R antagonist, exendin-(9-39) (0.5 *µ*mol/l; Sigma-Aldrich) (14). The islets were pre-treated with 100 nmol/l liraglutide, 5 mmol/l NAC or 0.5 *µ*mol/l exendin-(9-39) for 2 h followed by exposure to 0.5 mmol/l palmitate. The active GLP-1 content in the cell culture medium and cell lysates was measured using an ELISA kit (Linco Research).

### MTT assay

After functional analysis, 3-(4,5-dimethylthiazol-2-yl)-2,5-diphenyltetrazolium bromide (MTT) assay was used to determine the proportion of viable cells in the treated group compared to the control group, as previously described (23). The islets were incubated with 500 *µ*g/ml MTT in RPMI-1640 medium for 3 h at 37°C. At the end of the incubation period, the medium containing MTT was removed, and the islets or cells were dissolved in dimethyl sulfoxide (DMSO). The absorbance was measured at 540 and 690 nm using a microplate reader (Perkin Elmer EnSpire; Perkin Elmer, Waltham, MA, USA).

### Apoptosis assay

DNA fragmentation activity in the islets was quantified using a Cell Death Detection ELISA Plus kit (Roche Diagnostics GmbH, Mannheim, Germany) according to the manufacturer\s instructions. Following treatment, the cells were washed twice with PBS and incubated with lysis buffer for 20 min at room temperature. Following centrifugation to remove the nuclei and cellular debris, the supernatants were diluted 1:5 with lysis buffer, and each sample was analyzed using ELISA.

### Mmeasurement of intracellular levels of reactive oxygen species (ROS)

The quantification of intracellular ROS levels was carried out using the fluorescent probe, 2,7-dichlorofluorescein diacetate (DCFH-DA), as described in our previous study (24). The cells were washed twice with PBS and incubated in culture medium containing 20 *µ*mol/l DCFH-DA (Beyotime Institute of Biotechnology, Beijing, China) for 20 min at 37°C in the dark. Subsequently, the cells were lysed with lysis buffer, and the carboxy-DCF fluorescence in the cell lysates was measured using a multimode microplate reader (Bio-Rad, Hercules, CA, USA) and excitation and emission wavelengths of 488 and 530 nm, respectively.

### Immunoblot analysis

The islets were seeded into 24-well plates; 50 islets were added to each well. Following treatment, the islets were washed with PBS prior to the addition of cell lysis buffer containing protease inhibitor cocktail and PhoshopStop tablets (Roche Applied Science). The primary antibodies used were PC1/3 (1:1,000; AB10553; Millipore) and pancreatic duodenal homeobox 1 (PDX1) (1:1,000; 5679; Cell Signaling Technology, Boston, MA, USA). An antibody against mouse β-actin (A1978) was obtained from Sigma-Aldrich. Densitometric analysis was performed using ImageJ software.

### Immunodetection

After the mice were euthanized, and their pancreata were removed, the pancreatic tissue was harvested and fixed in 4% formaldehyde overnight and stored in 70% ethanol. Fixed sections of pancreatic tissues were embedded in paraffin and cut into 5-*µ*m-thick sections. Paraffin-embedded sections were rehydrated, and antigen retrieval was performed using a PickCell pressure cooker. The primary antibodies used were guinea pig anti-insulin (1:150; ab7842; Abcam, Cambridge, UK), rabbit anti-PC1/3 (1:200; Millipore), rabbit anti-glucagon (1:200; 8233; Cell Signaling Technology) and rabbit anti-NF-κB p65 (1:200, ab16502; Abcam). The secondary antibodies were conjugated to Alexa Fluor 488 [1:200; AffiniPure goat anti-guinea pig IgG (H+L); Cat. no. 106-545-003; Jackson ImmunoResearch Laboratories, West Grove, PA, USA] or DyLight 549 [1:200; goat anti-rabbit IgG (H+L); Cat. no. A23320; Abbkine Inc., Redlands, CA, USA]. The nuclear counterstain, 4′6′-diamidino-2-phenylindole (DAPI; Invitrogen, Carlsbad, CA, USA), was also used. All the digital images were acquired using a fluorescence microscope equipped with a DC200 digital camera (C-1/TE200U; Nikon, Tokyo, Japan) and were subsequently analyzed using Image-Pro Plus version 5.0 image analysis software (Media Cybernetics, Rockville, MD, USA). The density threshold selection tool was used to select the pancreatic islet areas marked with insulin and glucagon, which was depicted as a percentage of the mean islet cross-sectional area (immuno-density), as previoulsy descibed (25).

### Analyses of mRNA expression by quantitative polymerase chain reaction (qPCR)

Total RNA from the isolated islets and mouse pancreatic tissue samples was extracted using TRIzol reagent (Invitrogen). First-strand cDNA synthesis was performed using a cDNA synthesis kit (Takara Shuzo Co., Ltd., Kyoto, Japan) and qPCR was performed using a LightCycler (Roche Diagnostics GmbH). The primers used in this study are listed in Table I. The relative transcript levels were normalized to 36B4 and calculated using the 2^−ΔΔCT^ statistical method.

### Statistical analysis

The results of the present study are expressed as the means ± SEM values, and data were analyzed using the Student’s t-test or one-way analysis of variance (ANOVA) followed by the Bonferroni post-hoc test. A value of p<0.05 was considered to indicate a statistically significant difference.

## Results

### Activation of the endogenous GLP-1 system in injured isolated islets

**Figure 1:**
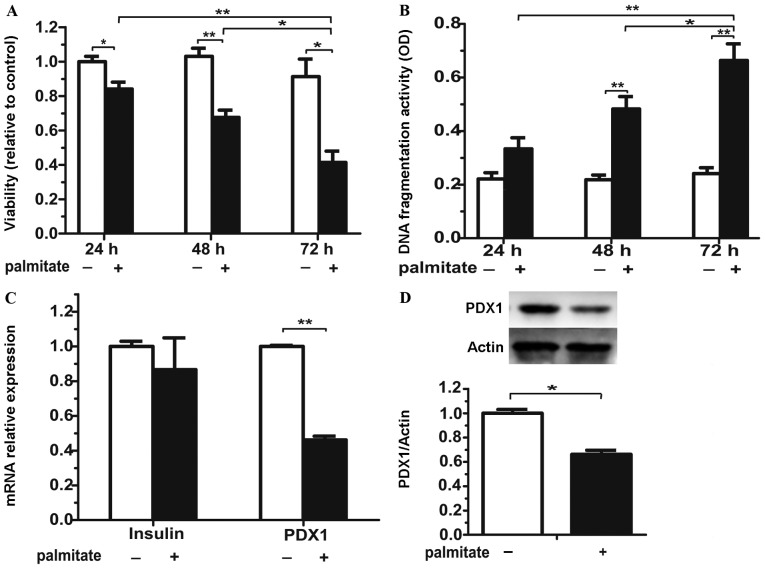
Palmitate induces injury to isolated mouse islets. Following incubation with 0.5 mmol/l palmitate for the indicated periods of time (24, 48 and 72 h), islet (A) viability and (B) apoptosis were analyzed by examining histone-associated DNA fragments and by MTT assay, respectively. Following incubation with 0.5 mmol/l palmitate for 48 h, (C) the insulin mRNA and (D) pancreatic duodenal homeobox 1 (PDX1) mRNA and protein levels were determined by qPCR and immunoblot analysis, respectively. The relative transcript levels were normalized to those of 36B4. n=3 separate islet preparations; ^*^p<0.05; ^**^p<0.01.

Following the incubation of the isolated mouse islets for 24, 48 or 72 h, treatment with 0.5 mmol/l palmitate for24, 48 and 72 h led to a significant decrease in islet viability and an increase in cell death in a time-dependent manner (Fig. 1A and B). Since the exposure of the cultured islets for 72 h was severely damaging, we focused on the effects of palmitate on β cell-specific transcription factors after 48 h of treatment. Incubation with palmitate for 48 h did not significantly modify the mRNA expression of insulin (Fig. 1C). Nonetheless, both PDX1 mRNA and protein (Fig. 1D) levels decreased after 48 h of exposure to palmitate. Thus, prolonged exposure to palmitate induces injury to isolated islets.

**Figure 2:**
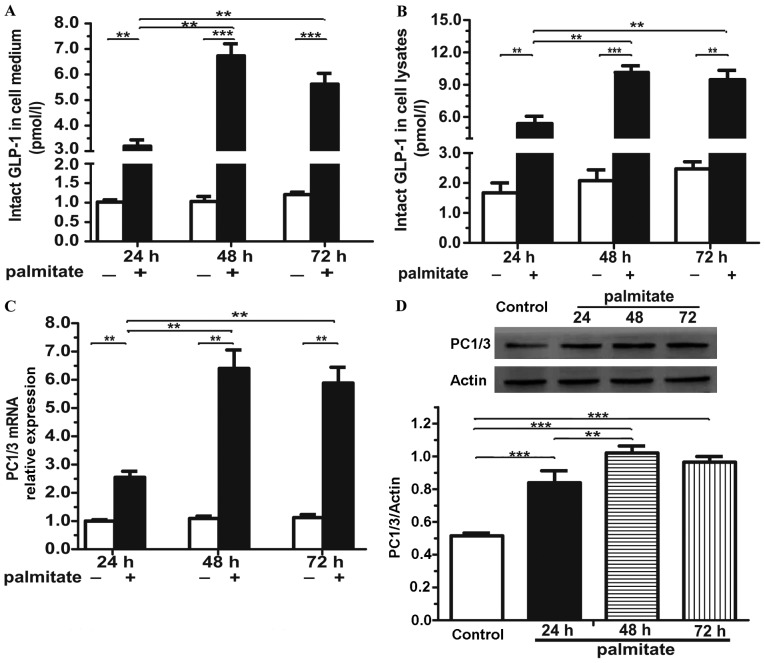
Prolonged exposure to palmitate induces the activation of the glucagon-like peptide-1 (GLP-1) system in isolated mouse islets. After the islet cells were incubated with 0.5 mmol/l palmitate for the indicated periods of time, the GLP-1 levels in the (A) cell medium and (B) lysates were determined by insulin enzyme-linked immunosorbent assay (ELISA), and (C) prohormone convertase 1/3 (PC1/3) mRNA expression in cell lysates was determined by qPCR and (D) the PC1/3 protein level was determined by immunoblot anlaysis. n=3 separate islet preparations; ^**^p<0.01; ^***^p<0.001.

Importantly, incubation of the cells with 0.5 mmol/l palmitate for 24, 48 or 72 h markedly induced the release of GLP-1 into the culture medium by 3.15-, 6.55- and 5.62-fold, respectively (Fig. 2A). Moreover, in line with the observations of the culture medium, the GLP-1 concentration in the cell lysates was elevated and showed an even greater increase (Fig. 2B). Nonetheless, the GLP-1 levels at 72 h in both the cell medium and the cell lysates were almost equivalent to those at 48 h and even showed a decreasing trend (Fig. 2A and B), which may be due to the severe serious injury induced by 72 h of exposure to palmitate (Fig. 1A and B; increased apoptosis and decreased cell viability). Following prolonged exposure to palmitate, the mRNA (Fig. 2C) and protein levels (Fig. 2D) of PC1/3, the key enzyme of GLP-1 generation, also increased (48 h of exposure resulted in higher mRNA and protein levels than 72 h of exposure).

### Elevated expression of GLP-1 in local pancreatic islets in vivo

**Figure 3:**
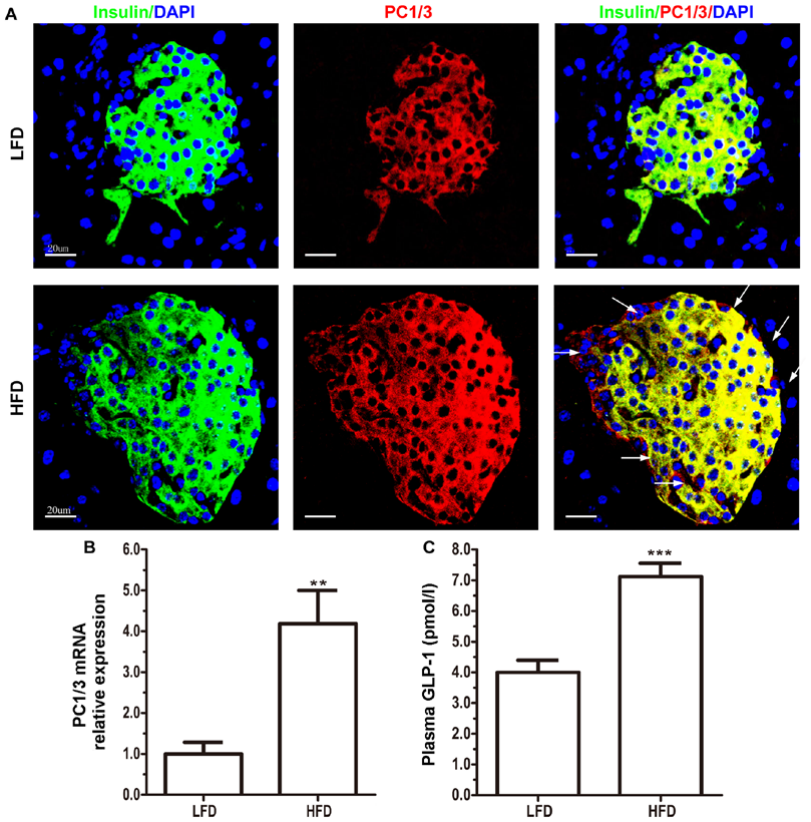
High-fat diet (HFD) induces intra-islet glucagon-like peptide-1 (GLP-1) system activation. (A) Representative images of immunofluorescence staining for insulin (green) and prohormone convertase 1/3 (PC1/3) (red) in the pancreatic islets of mice that were fed a low-fat diet (LFD) (n=6) or a HFD (n=7) for 8 weeks. The arrows indicate the insulin-negative and PC1/3-positive cells. (B) PC1/3 mRNA expression in the pancreata of mice from each group was detected by qPCR. (C) Plasma GLP-1 levels in the mice were detected by insulin enzyme-linked immunosorbent assay (ELISA). LFD compared with HFD; ^**^p<0.01; ^***^p<0.001.

After 8 weeks of being fed a HFD, PC1/3 protein expression in the HFD group markedly increased (Fig. 3A). Normally, insulin co-localizes with PC1/3 (Fig. 3A), which is known to cleave pro-insulin at the B-chain/C-peptide (26). However, we identified a few PC1/3-positive cells in the extracellular β cell compartments (Fig. 3A). When the PC1/3-positive and insulin-negative α cells were quantified, an increased number of these cells (163 cells) was found in the HFD group (n=7) compared with the LFD group (n=6; 48 cells) in every 20 slices (data not shown). In addition to intestinal L cells, PC1/3, the key enzyme governing GLP-1 formation, is expressed in islet β cells and pro-α cells (27), suggesting that the elevated number of PC1/3-positive and insulin-negative cells may be pro-α cells. Notably, in the HFD group, pancreatic PC1/3 mRNA expression was upregulated (Fig. 3B), and the plasma GLP-1 concentrations were increased compared with the LFD group (Fig. 3C), which was in accordance with the increase in PC1/3 protein expression (Fig. 3A) and may be the result of the activation of the intra-islet GLP-1 system. These results demonstrated that a HFD also induced the formation of pro-α cells and the release of GLP-1 through the upregulation of the expression of PC1/3.

### Inhibition of GLP-1R signaling exacerbates the detrimental effects of palmitate

**Figure 4:**
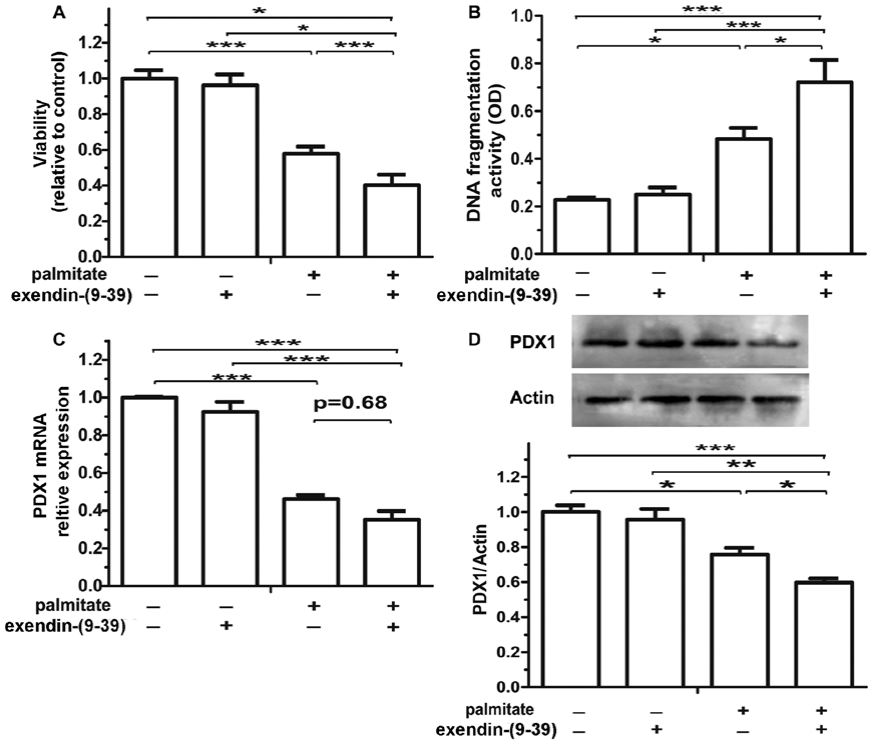
Glucagon-like peptide-1 receptor (GLP-1R) inhibition exacerbates the detrimental effects of palmitate. Isolated islets were pre-treated with the GLP-1R antagonist, exendin-(9-39), (0.5 *µ*mol/l) for 2 h, followed by exposure to 0.5 mmol/l palmitate for 48 h. Islet (A) viability (detected by MTT assay) and (B) apoptosis (determined by examining histone-associated DNA fragments), and pancreatic duodenal homeobox 1 (PDX1) (C) mRNA (detected by qPCR) and (D) protein levels (detected by immunoblot analysis). n=3 separate islet preparations; ^*^p<0.05; ^**^p<0.01; ^***^p<0.001.

Treatment with exendin-(9-39) alone, another GLP-1-derived peptide and a GLP-1R antagonist, did not exert any effects on the isolated islets. When combined with exposure to palmitate for 48 h, treatment with exendin-(9-39) resulted in the progressive loss of islet cells that exhibited decreased viability (Fig. 4A) and higher apoptotic levels (Fig. 4B). Furthermore, treatment with exendin-(9-39) exacerbated the detrimental effects of palmitate on β cell survival by decreasing PDX1 mRNA (Fig. 4C) (p=0.68) and protein expression (Fig. 4D). These results suggest that the activation of the intra-islet GLP-1 system ameliorates the detrimental effects of palmitate.

### GLP-1R agonist attenuates lipotoxicity-induced islet dysfunction in vitro and in vivo

**Figure 5:**
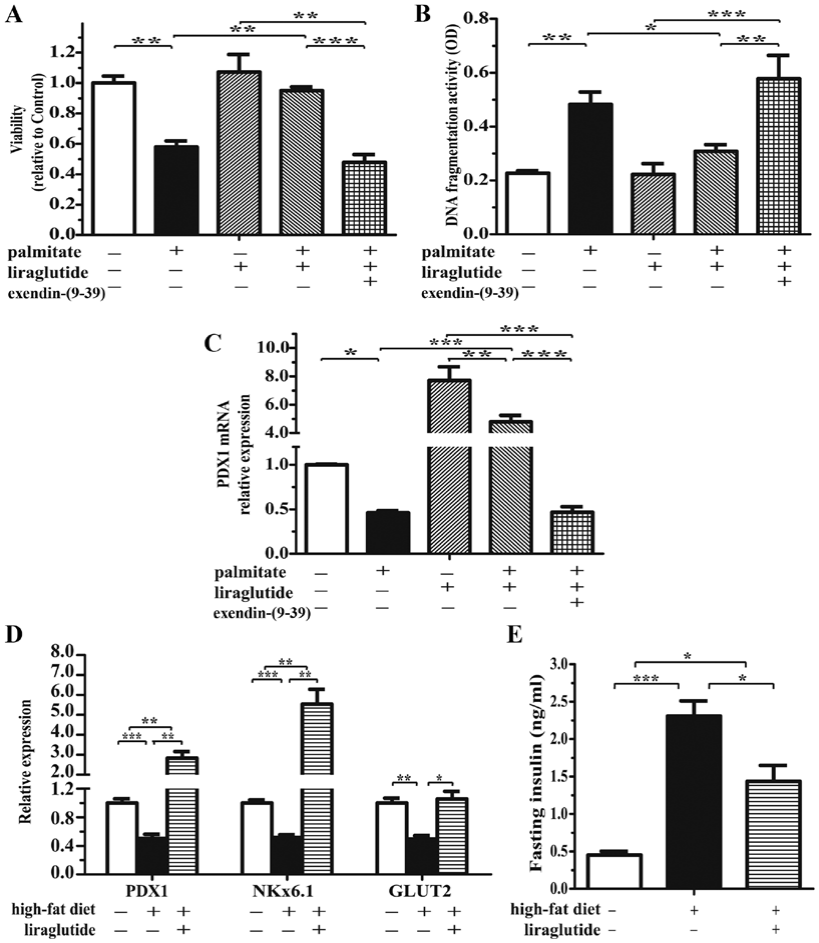
The glucagon-like peptide-1 receptor (GLP-1R) signaling agonist, liraglutide, attenuates lipotoxicity-induced islet dysfunction. Isolated non-diabetic islets were pre-treated with 100 nmol/l liraglutide and/or 0.5 *µ*mol/l exendin-(9-39) for 2 h, followed by exposure to 0.5 mmol/l palmitate for 48 h. Islet (A) viability (detected by MTT assay) and (B) apoptosis (determined by examining histone-associated DNA fragments), and (C) pancreatic duodenal homeobox 1 (PDX1) mRNA levels (detected by qPCR). n=3 separate islet preparations. (D) After 8 weeks on their respective diets, the mRNA expression of PDX1, glucose transporter 2 (GLUT2) and Nkx6.1 was detected in the pancreata of the mice. (E) The plasma insulin levels were detected by insulin enzyme-linked immunosorbent assay (ELISA). n=6–7 mice/group; ^*^p<0.05; ^**^p<0.01; ^***^p<0.001.

Considering the short biological half-life of exendin-(9-39) [Kieffer *et al* (28)], we used the stable, long-lived GLP-1 analog, liraglutide, which is an agonist of GLP-1R. Intriguingly, compared to treatment with palmitate alone, treatment with liraglutide significantly increased islet viability (Fig. 5A) and decreased islet cell apoptosis (Fig. 5B) to levels close to those of the controls. Liraglutide also markedly upregulated PDX1 mRNA expression by 7.70-fold, and this increase was attenuated by treatment with palmitate (Fig. 5C). Moreover, the protective effects of liraglutide were completely abrogated by exendin-(9-39) (Fig. 5A–C). We also investigated the effects of liraglutide on islet function in mice fed a HFD. In the HFD group, liraglutide also increased the mRNA expression of the β cell markers, PDX1, Nkx6.1 and glucose transporter 2 (GLUT2) (Fig. 5D). Compared to the mice fed a HFD alone, treatment with liraglutide reduced the plasma insulin concentration (Fig. 5E).

### Liraglutide normalizes the islet architecture of mice fed a HFD

**Figure 6:**
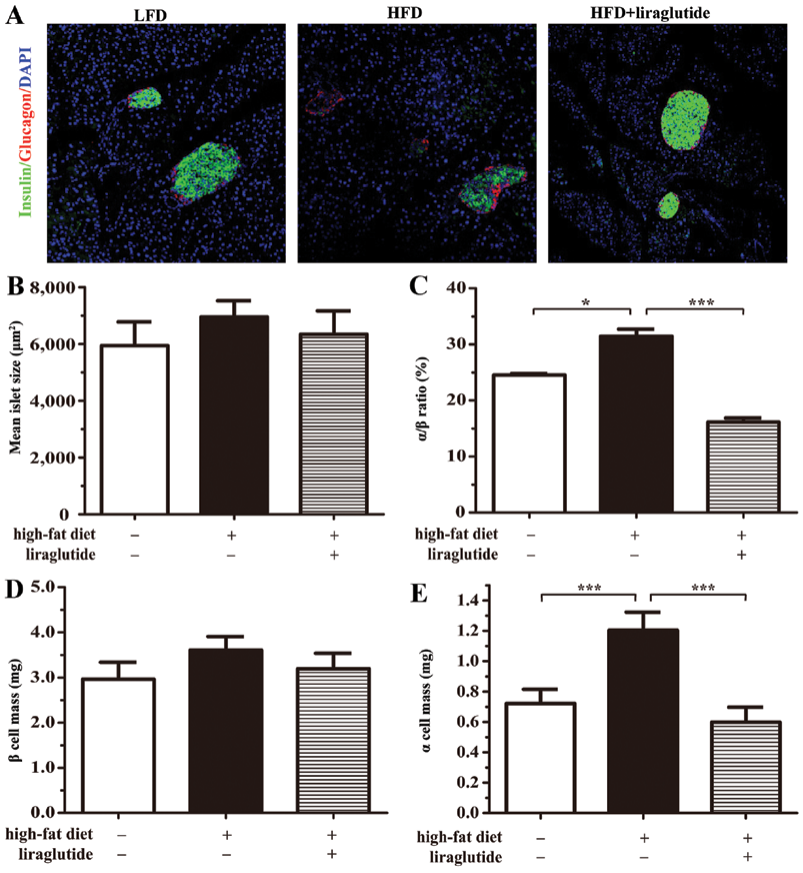
Liraglutide normalizes the islet architecture of mice fed a high-fat diet (HFD). (A) Representative images of staining for insulin (green) and glucagon (red). After immunofluorescence, areas labeled for insulin (β-cell) and glucagon (red) were measured, and the results are expressed as the mean (B) islet size, (C) α/β ratio, (D) β cell mass and (E) α cell mass. Analyses were performed on histological sections obtained from mice fed a low-fat diet (LFD) + the placebo (phosphate-buffered saline) (LFD; n=6), a HFD + placebo (phosphate-buffered saline) (HFD; n=7) or a HFD + liraglutide (n=6). ^*^p<0.05; ^***^p<0.001.

Unlike the defined α cell mantle and β cell core characteristics of the islets from the mice fed a LFD, the islets from the mice fed a HFD maintained a more scattered organization and a higher percentage of α cells. Furthermore, in the HFD group, there was a greater difference in the expression of insulin (Fig. 6A). As expected, treatment with liraglutide reverted the cell structure to a more normal islet structure (Fig. 6A). Despite the differences in the mean islet areas of the 3 groups (Fig. 6B), the elevated proportions of medium islets (5,000–10,000 *µ*m^2^) and large islets (>5,000 *µ*m^2^), as well as the mean area of small islets (<5,000 *µ*m^2^) that were induced by a HFD were altered by treatment with liraglutide (Table II). In accordance with an elevated α/β cell ratio, the HFD group exhibited a significant increase in α cell mass (Fig. 6C and E). Treatment with liraglutide decreased the β cell mass (Fig. 6D). However, these results do not completely agree with previously published findings (29), possibly due to the different experimental conditions used, the increased β cell proportion and the inhibitory effects of a HFD on insulin expression.

### The excess production of ROS induced by palmitate activates the GLP-1 system

**Figure 7:**
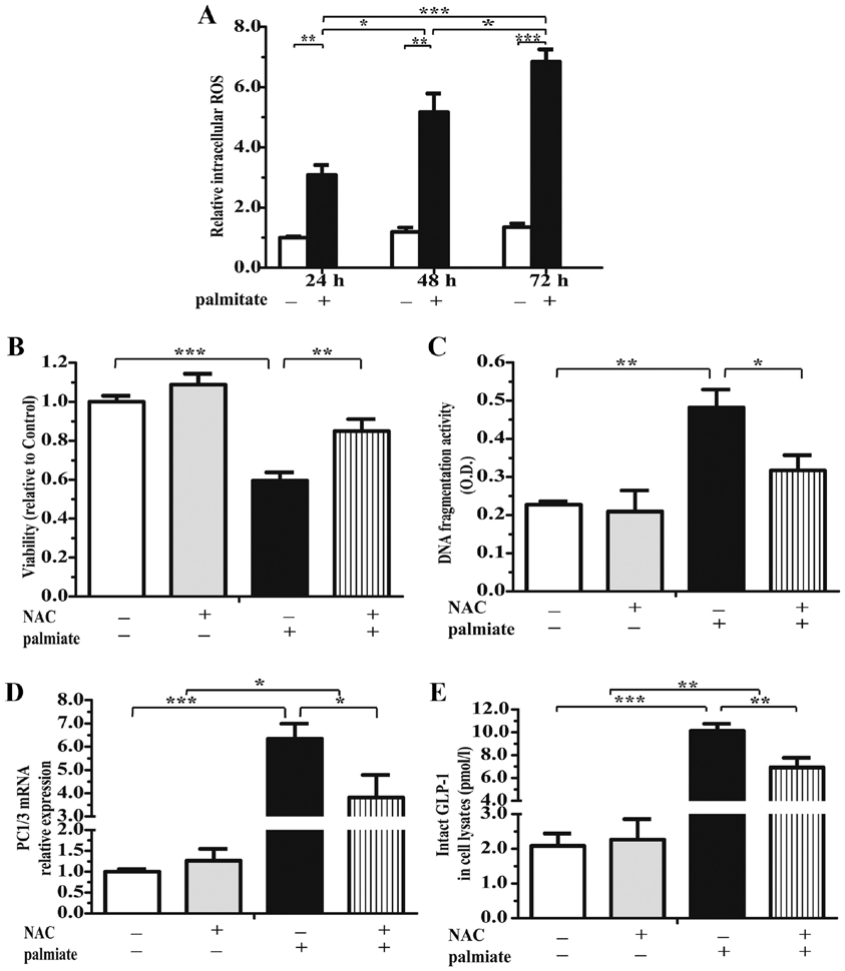
Prolonged exposure to palmitate induces reactive oxygen species (ROS) productin and activates the glucagon-like peptide-1 (GLP-1) system. (A) Following incubation with 0.5 mmol/l palmitate for the indicated periods of time, ROS levels were detected by DCFH-DA. Following pre-incubation with 5 mmol/l N-acetylcysteine (NAC) for 48 h, cell (B) viability (by MTT assay) and (C) apoptosis (by examining histone-associated DNA fragments), and (D) prohormone convertase 1/3 (PC1/3) mRNA (by qPCR) and (E) GLP-1 levels (by ELISA) in the cell lysates were determined. n=3 separate isolated islet preparations; ^*^p<0.05; ^**^p<0.01; ^***^p<0.001.

Incubation of the cells with 0.5 mmol/l palmitate for 24, 48 or 72 h markedly increased the intra-islet ROS levels by approximately 3.01-, 5.12- and 6.45-fold, respectively (Fig. 7A). In addition, pre-incubation with 5 mmol/l NAC, neutralized the harmful effects of palmitate, increased islet viability (by 1.43-fold; Fig. 7B) and decreased apoptosis by approximately 64.84% (Fig. 7C) in the presence of 0.5 mmol/l palmitate. Importantly, NAC significantly decreased the PC1/3 mRNA levels (Fig. 7D) and the GLP-1 concentration in the cell lysates (Fig. 7E), which may be due to the attenuation of β cell injury. Nonetheless, the elevated levels of PC1/3 mRNA (Fig. 7D) and GLP-1 protein, which were induced by palmitate, did not return to normal after NAC treatment (Fig. 7E), suggesting that the inhibition did not completely reverse the detrimental effects of palmitate overload in the pancreatic islets.

### GLP-1R signaling helps to maintain the oxidative balance

**Figure 8:**
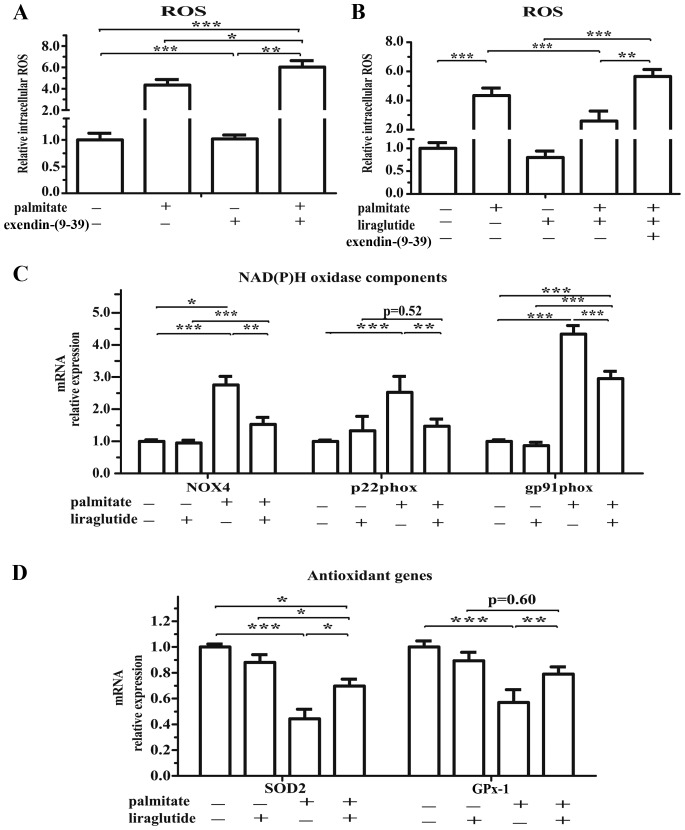
Glucagon-like peptide-1 receptor (GLP-1R) signaling exerts inhibitory effects on the generation of reactive oxygen species (ROS). (A and B) Following pre-incubation with 100 nmol/l liraglutide and/or 0.5 *µ*mol/l exendin-(9-39) for 2 h, followed by exposure to 0.5 mmol/l palmitate for 48 h, ROS levels were detected byDCFH-DA, and (C) the mRNA expression of NAD(P)H oxidase components [including NADPH oxidase 4 (NOX4), p22phox and gp91phox], and (D) antioxidant genes [superoxide dismutase 2 (SOD2) and glutathione peroxidase-1 (GPx-1)] was detected by qPCR. n=3 separate isolated islet preparations; ^*^p<0.05; ^**^p<0.01; ^***^p<0.001.

Given the intermediary role of oxidative stress in palmitate-induced injury, we hypothesized that GLP-1R signaling may re-shape the oxidative balance by suppressing the generation of ROS and enhancing antioxidant defenses. The inhibition of GLP-1R signaling by exendin-(9-39) (in combination with palmitate) also slightly increased the production of ROS (p=0.045; Fig. 8A). Furthermore, the activation of GLP-1R by liraglutide decreased the ROS levels in the isolated islets (p=0.038; Fig. 8B). Notably, these changes were directly confirmed by qPCR, which revealed a clear and widespread reshaping of the oxidative balance. Treatment with liraglutide attenuated the palmitate-induced activation of NAD(P)H oxidase components, including NADPH oxidase 4 (NOX4), p22phox and gp91phox (Fig. 8C). Simultaneously, treatment with liraglutide upregulated the expression of mitochondrial-specific superoxide dismutase 2 (SOD2) and glutathione peroxidase 1 (GPx-1) (Fig. 8D).

### Liraglutide helps to attenuate islet inflammation

**Figure 9:**
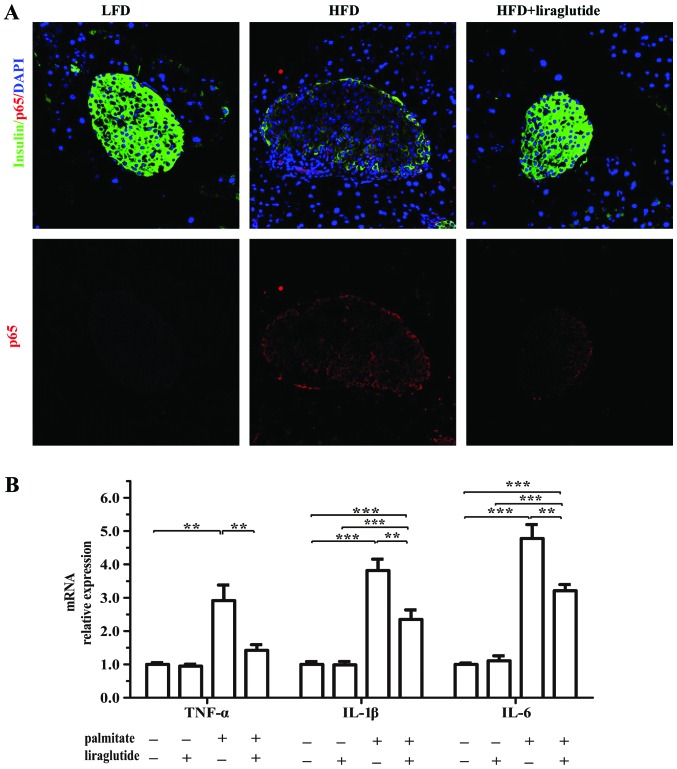
Glucagon-like peptide-1 receptor (GLP-1R) signaling suppresses islet inflammation. (A) Representative images of staining for insulin (green) and p65 (red) *in vivo*. n=6–7 mice. (B) Following pre-incubation with 100 nmol/l liraglutide for 2 h, followed by exposure to 0.5 mmol/l palmitate for 48 h, tumor necrosis factor-α (TNF-α), interleukin (IL)-1β and IL-6 mRNA levels were detected by qPCR. n=3 separate isolated islet preparations. ^**^p<0.01; ^***^p<0.001.

Based on the fact that the nuclear factor-κB (NF-κB) activation by ROS (30) and inflammation are key to the development of β cell failure (31), we examined the effects of liraglutide on inflammatory factors and the NF-κB pathway in pancreatic islets. Double immunostaining revealed that the p65 protein expression levels were markedly increased in the pancreatic islets of the mice fed a HFD compared to those of the islets of the mice fed a LFD (Fig. 9A). Furthermore, in the isolated islets, treatment with liraglutide suppressed the palmitate-induced expression of inflammatory factors, including tumor necrosis factor-α (TNF-α), interleukin (IL)-1β and IL-6 (Fig. 9B).

## Discussion

To the best of our knowledge, the present study for the first time examined the hypothesis that lipotoxicity directly stimulates the generation of immature pro-α cells, resulting in pro-α cells producing endogenous GLP-1 to facilitate β cell survival by maintaining the oxidative balance and by inhibiting islet inflammation. Prolonged exposure to palmitate induced lipotoxicity, and a HFD induced PC1/3 expression and, in turn, increased the synthesis and release of GLP-1, which were partly mediated by lipotoxicity-induced oxidative stress. The activation of GLP-1R signaling was attributed to the normalization of islet function and structure. Furthermore, GLP-1 exerted protective effects against lipid overload, partially by increasing antioxidant gene expression and decreasing the levels of ROS, NF-κB and inflammatory factors.

Evidence examining the striking innate plasticity of islets has recently received significant attention as the dedifferentiation of hyperplastic α cells maintains an immature pro-α phenotype in response to β cell stress or injury (16). As α cells constitutively express proglucagon, their plasticity is manifested in the expression of glucagon or GLP-1 and depends on the relative levels of PC2 and PC1/3 (16). In models of insulin resistance or diabetes, there is a significant, progressive increase in intra-islet GLP-1 expression based on the upregulation of PC1/3 expression (32,33). The present study similarly demonstrated that lipotoxicity upregulated GLP-1 expression in response to β cell injury. Generally, the increase in GLP-1 expression was attributed to pro-α cells, which are derived from the dedifferentiation of hyperplastic α cells. β cells may serve as an alternative source of pro-α cells since the dedifferentiation of β cells to progenitor-like cells caused by metabolic stress may also represent a distinct ‘pro-α’ cell differentiation stage (34,35). Therefore, the identification of a set of pro-α cells that contribute to the endogenous GLP-1 system supports the view that it is advantageous for islet cells to dedifferentiate to facilitate their survival.

Unequivocally, in our study, intra-islet GLP-1 enhanced β cell survival against lipotoxicity through a pleiotropic mechanism. Initially, intra-islet GLP-1/GLP-1R signaling partially decreased the palmitate-induced damage to β cells. The GLP-1R antagonist, exendin-(9-39), further suppressed islet viability, increased apoptosis and inhibited PDX1 transcription in the presence of palmitate after 48 h. Additionally, our data demonstrated that GLP-1/GLP-1R signaling attenuated the transcription of β cell markers, such as insulin, PDX1, Nkx6.1 and GLUT2. GLP-1R signaling upregulates the insulin gene promoter by inhibiting p38 mitogen-activated protein kinase (p38 MAPK) (8). The shielding action of GLP-1 also involves the activation of the phosphoinositide 3-kinase (PI3K) and extracellular signal-regulated kinase (ERK) pathways and the upregulation of PDX-1 transcription (36). Finally, GLP-1 normalizes islet structure by promoting proliferation, inhibiting apoptosis and normalizing the distribution of islet cells. GLP-1 protects pancreatic β cells against lipotoxicity-induced apoptosis by activating peroxisome proliferator-activated receptor (PPAR)-β/δ (7) and promotes β cell proliferation mediated through the EGF pathway (5). Therefore, the activation of the endogenous GLP-1 system appears to be a ‘self-defense’ response in pancreatic islets. However, whether there are other more specific ‘protectors’ in the prevention and treatment of lipotoxicity remains to be determined.

Our data suggest that oxidative stress plays a role as the bridge between lipid overload and the intra-islet GLP-1 system. Oxidative stress is a common biochemical trigger of stress-sensitive signaling pathways, including NF-κB, p38 MAPK and JNK (37,38). Moreover, β cells are particularly susceptible to the damage inflicted by oxidative stress due to low levels of free-radical scavenging enzymes (37,39). Exendin-4, another GLP-1R agonist, protects endothelial cells from palmitate-induced apoptosis by modulating stress-sensitive signaling pathways (40). In a previous study, in rats with tacrolimus-induced diabetes, the dipeptidyl peptidase-4 (DPP4) inhibitor, MK-0626, decreased the levels of 8-OHdG (a marker of oxidative DNA damage) and increased the levels of manganese superoxide dismutase and heme oxygenase-1 (41). In rats with streptozotocin-induced diabetes, liraglutide was shown to directly protect the rats against oxidative stress through the inhibition of NAD(P)H oxidases (11). Consistent with our results, Lotfy *et al* (9) reported that another GLP-1R signaling agonist, exenatide, elevated the expression of catalase and glutathione reductase in the pancreata of diabetic rats. Small GLP-1-derived peptides also modulate nutrient homeostasis by suppressing oxidative stress (42,43). In addition, ROS activate NF-κB and induce the generation of inflammatory factors, and our data, as well as previous findings demonstrate that liraglutide decreases p65 expression and the expression of inflammatory factors, such as TNF-α and IL-1β (30).

In conclusion, the findings of the present study suggest that endogenous GLP-1/GLP-1R signaling is a ‘self-defense’ pathway that facilitates islet survival against lipotoxicity. The protective mechanism of intra-islet GLP-1 may be the achievement of oxidative balance, as well as the inhibition of islet inflammation. Further research on the intra-islet GLP-1 system is required to obtain a better understanding of the mechanisms through which adult β cells maintain or change their identity due to lipotoxicity.

## Figures and Tables

**Table I:** List of primers used for qPCR using SYBR-Green.

Gene	Gene ID	Forward sequence (5′→3′)	Reverse sequence (5′→3′)	Length
Nkx6.1	NM_144955.2	ACTTGGCAGGACCAGAGAG	GCGTGCTTCTTTCTCCACTT	109
PDX1	NM_008814.3	TGAACTTGACCGAGAGACACAT	GGTCCCGCTACTACGTTTCTTA	92
PC1/3	NM_013628.2	ACATGGGGAGAGAATCCTGTAGGCA	CATGGCCTTTGAAGGAGTTCCTTGT	220
Insulin-1	NM_008386.3	GGACCCACAAGTGGAACAAC	GCTGGTAGAGGGAGCAAATG	130
GLUT2	NM_031197.2	GCCAAGTAGGATGTGCCAAT	CCCTGGGTACTCTTCACCAA	110
NOX4	NM_015760.5	ATTTGGATAGGCTCCAGGCAAAC	CACATGGGTATAAGCTTTGTGAGC	155
p22phox	NM_001301284.1	GGCACCATCAAGCAACCACC	CTCATCTGTCACTGGCATTGGG	135
gp91phox	NM_007807.5	TCCGTATTGTGGGAGACTGGACG	AATGGAGGCAAAGGGCGTGAC	194
SOD2	NM_013671.3	CAGACCTGCCTTACGACTATGG	CTCGGTGGCGTTGAGATTGTT	113
GPx-1	NM_008160.6	CCTCAAGTACGTCCGACCTG	CAATGTCGTTGCGGCACACC	197
IL-1β	NM_008361.3	GCACACCCACCCTGCA	ACCGCTTTTCCATCTTCTTCTT	69
IL-6	NM_031168.1	TCCAGAAACCGCTATGAAGTTC	CACCAGCATCAGTCCCAAGA	73
TNF-α	NM_013693.3	CTCCAGGCGGTGCCTATG	GGGCCATAGAACTGATGAGAGG	149
36B4	NM_007475.5	CAGCAAGTGGGAAGGTGTAATCC	CCCATTCTATCATCAACGGGTACAA	75

Length is indicate in bp. PDX1, pancreatic duodenal homeobox 1; PC1/3, prohormone convertase 1/3; GLUT2, glucose transporter 2; NOX4, NADPH oxidase 4; SOD2, superoxide dismutase 2; GPx-1, glutathione peroxidase 1; TNF-α, tumor necrosis factor-α; IL, interleukin.

**Table II:** The percentage and mean area (*µ*m^2^) of small, medium and large-sized islets per group.

	LFD		HFD		HFD + liraglutide	
	Percentage	Mean area	Percentage	Mean area	Percentage	Mean area
Small islets (<5,000 *µ*m^2^)	70.37	1470.86±115.034	54.40	2122.65±154.12^a^	66.67	2027.76±148.49^a^
Medium islets (5,000–10,000 *µ*m^2^)	15.43	7696.40±256.16	25.82	7562.77±209.21	20.60	8101.04±256.92
Large islets (>10,000 *µ*m^2^)	14.20	26220.00±3448.25	19.78	19460.94±1306.30	12.73	26757.57±3244.72

Results are means ± SEM (n=6–7).

a p<0.05 vs. control. LFD, low-fat diet; HFD, high-fat diet.
